# Association of rs944289, rs965513, and rs1443434 in TITF1/TITF2 with Risks of Papillary Thyroid Carcinoma and with Nodular Goiter in Northern Chinese Han Populations

**DOI:** 10.1155/2020/4539747

**Published:** 2020-02-11

**Authors:** Xin Zhang, Yulu Gu, Yong Li, Heran Cui, Xiaoli Liu, Hui Sun, Qiong Yu, Yaqin Yu, Yawen Liu, Siyan Zhan, Yi Cheng

**Affiliations:** ^1^Department of Epidemiology and Biostatistics, School of Public Health, Jilin University, Changchun 130021, China; ^2^Department of Pharmacy, The First Hospital of Jilin University, Changchun 130021, China; ^3^Jilin Provincial Key Laboratory of Surgical Translational Medicine, Department of Thyroid and Parathyroid Surgery, China-Japan Union Hospital, Jilin University, Changchun 130033, China; ^4^Department of Epidemiology and Biostatistics, School of Public Health, Peking University Health Science Centre, Beijing 100191, China; ^5^Department of Cardiovascular Center, First Hospital of Jilin University, Changchun 130021, China

## Abstract

**Objective:**

In this study, we aimed to investigate the associations of three single-nucleotide polymorphisms (SNPs) on TITF1/TITF2 (rs944289, rs965513, and rs1443434) with susceptibility to papillary thyroid carcinoma (PTC) and with nodular goiter (NG) in northern Chinese Han populations.

**Methods:**

We performed a case-control study comprising 861 PTC patients, 562 NG patients, and 896 normal controls (NCs). One TITF1 SNP (rs944289) and two TITF2 SNPs (rs965513 and rs1443434) were genotyped. Departures from Hardy–Weinberg equilibrium (HWE) in the control group were evaluated using chi-square test. Associations of the SNPs with PTC and with NG were assessed by unconditional logistic regression using the online SNPStats program. Bonferroni correction was performed for multiple tests in genotype analyses. Data analysis was performed by SPSS24.0 unless otherwise specified.

**Results:**

For rs944289, T allele was associated with increased risks for both PTC (OR = 1.23, 95% CI: 1.08–1.41, *P*=0.002) and NG (OR = 1.28, 95% CI: 1.10–1.50, *P*=0.002) and NG (OR = 1.28, 95% CI: 1.10–1.50, *P*=0.002) and NG (OR = 1.28, 95% CI: 1.10–1.50, *P*=0.002) and NG (OR = 1.28, 95% CI: 1.10–1.50, *P*=0.002) and NG (OR = 1.28, 95% CI: 1.10–1.50, *P*=0.002) and NG (OR = 1.28, 95% CI: 1.10–1.50, *P*=0.002) and NG (OR = 1.28, 95% CI: 1.10–1.50, *P*=0.002) and NG (OR = 1.28, 95% CI: 1.10–1.50,

**Conclusions:**

There are associations of rs944289 and rs1443434 polymorphisms with PTC risk and association of rs944289 polymorphism with NG risk. Haplotypes T-G-G and T-G-T are risk haplotypes of PTC and NG, respectively.

## 1. Introduction

Thyroid cancer, as one of the most common tumor of endocrine system, is the fifth leading cancer for the estimated new cancer cases in females [1, 2]. Among all the histological subtypes of thyroid cancer, papillary thyroid carcinoma (PTC) is the most common type, accounting for about 75–85% of all cases with thyroid cancer.

Genetic factors are essential for the pathogenesis of thyroid cancer [3]. Thyroid cancer has the highest heritability (53%) among 15 common cancers, with very low shared environmental effects (1%) [4]. A joint study from five Nordic countries revealed that lifetime cumulative risk of female relatives of PTC patients (2%) is higher than that of males (1%); the more PTC patients a family has, the more risk of thyroid cancer their relatives has; and cumulative risk of thyroid cancer increases threefold in comparison with general population lifetime [5]. Moreover, a study from the Korea National Health and Nutrition Examination Surveys showed that factors related to thyroid function, such as thyroid-stimulating hormone (TSH) and free thyroxine (fT4), also present high heritability: the narrow-sense heritability for TSH is 54%, and that for free thyroxine (fT4) is 56%; the narrow-sense heritability is higher in females than that in males for TSH; and both TSH and fT4 show negative genetic correlations in females after adjusted for environmental effects [6]. In a twin study including 69 monozygotic and 45 dizygotic adult twin pairs, the environment-adjusted heritability of the thickness of thyroid isthmus and thyroid volume (left and right lobes) on thyroid cancer was 50% and 68–79%, respectively [7]. These evidences reflect the high heritability of thyroid cancer.

Molecular mechanisms (gene mutation, metastasis suppressors abnormally expression, epigenetic modification, microRNA regulation, and signaling pathway alteration) are involved in thyroid cancer pathogenesis [8, 9]. The BRAF^V600E^ mutation and RAS mutation has been identified as the most prevalent oncogenic event in thyroid cancer [9]. Novel risk loci on different genes have further been identified and validated in different populations, providing a new dimension to this extensive line of thyroid cancer research [10, 11]. TITF1 and TITF2 are located on chromosome 14q13.3 and 9q22.33, respectively, encoding thyroid-specific transcription factors (TITF1 and TITF2) [12, 13]. TITF1 is a homeodomain-containing nuclear transcription factor, being crucial in thyroid development and differentiation by regulating thyroid-specific genes thyroglobulin (Tg), thyroperoxidase (TPO), and thyrotropin receptor (TSHR) [14]. TITF2 contains a forkhead domain and encodes a phosphorylated protein. Mutations in TITF2 lead to the Bamforth syndrome, penetrating as thyroid agenesis [15]. These indicate that important loci mutations on TITF1 and TITF2 may modify the expression of these two transcription factors and influence their function on thyroid gland. Recently, different single-nucleotide polymorphisms (SNPs) in TITF1 (also called NKX2-1 and TTF1)/TITF2 (also called FOXE1 and TTF2) are found in PTC, suggesting that rs944289, rs965513, and rs1443434 may be implicated in PTC risk [2, 13]. However, these studies are performed in populations with different ethnic background, conferring inconsistent results [16–18].

In this study, we evaluated the association of rs944289, rs965513, and rs1443434 in TITF1/TITF2 with PTC risks and with nodular goiter (NG) in northern Chinese Han populations.

## 2. Materials and Methods

### 2.1. Patients and Samples

A total of 861 patients with PTC, 562 patients with NG, and 896 normal controls (NCs) were included in this study. The patients with PTC and the patients with NG were recruited from January 2012 to December 2014 in the China-Japan Union Hospital of Jilin University, China. The inclusion criteria of PTC and NG patients included the followings: (1) northern Chinese Han populations; (2) pathologically diagnosed based on the Revised American Thyroid Association Management Guidelines for patients with thyroid nodules and differentiated thyroid cancer [19]; (3) histologic type of PTC was pure papillary carcinoma other than follicular variant of PTC (fvPTC). The NCs were selected from a large-scale community-based cross-sectional survey of chronic disease and risk factors among adults in Jilin province conducted in July 2012. All the NCs were northern Chinese Han populations in community with the following exclusion criteria: (1) with metabolic disease (hypertension, diabetes, and dyslipidemia), (2) with any thyroid disease (thyroiditis, benign and malignant thyroid tumors), (3) with other malignancy, and (4) with mental disorders. Demographic and clinical information of the patients and controls were obtained through inquiry or consulting medical records. Peripheral blood was provided by each participant for genotyping. The study was approved by the ethics committee of the School of Public Health, Jilin University, and written informed consents were obtained from the patients and controls in the study.

### 2.2. Selection of SNPs

We selected rs944289 in TITF1, and rs965513 and rs1443434 in TITF2 in this study based on previous genome-wide association studies (GWAS) and confirmatory studies [18, 20–22].

### 2.3. DNA Extraction and SNP Genotyping

Genomic DNA extraction and SNP genotyping were performed as in our previously published study [23]. Briefly, 5 ml of peripheral blood obtained from each patient and control was stored in EDTA-containing tubes. Genomic DNA was extracted using a commercial DNA extraction kit (ClotBlood DNA kit, Cwbio, Beijing, China) according to the manufacturer's instructions. Primers for polymerase chain reaction (PCR) were designed by Assay Designer 3.1 (Supplementary [Supplementary-material supplementary-material-1]). Genotypes were detected by matrix-assisted laser desorption/ionization time of flight mass spectrometry (MALDI-TOF-MS) using the MassARRAY system (Sequenom, San, Diego, CA, USA). The genotyping rate of rs944289, rs965513, and rs1443434 was 99.09%, 97.15%, and 95.34, respectively.

### 2.4. Statistical Analysis

Continuous variables were presented with mean and standard deviation, and categorical variables were presented with percentage. Differences between groups were tested on the basis of independent-samples *t*-test or chi-square test using SPSS Statistics 24.0 (IBM Canada Ltd.). Departure from Hardy–Weinberg equilibrium (HWE) in NCs was calculated on the basis of chi-square test using SPSS Statistics 24.0 (IBM Canada Ltd.). Associations between SNPs and PTC/NG under five models of inheritance (codominant, dominant, recessive, overdominant, and allele model) were performed using online SNPStats program (http://bioinfo.iconcologia.net/SNPStats) [24]. Strength of association was assessed by odds ratio (OR) and corresponding 95% confidence intervals (CIs). The best model of inheritance for each SNP was selected on the basis of the Akaike information criterion (AIC). Bonferroni correction was performed to reduce type I error in multiple testing in view of 15 comparisons between groups (three SNPs × five models); thus, significant *P* value was set at 0.05/15 = 0.003 for genotype analyses. Linkage disequilibrium analysis between the three SNPs and haplotype analysis was analyzed using the online SNPStats program. Power calculations were done using Quanto 1.2.4 software. Two-sided test with *P* value less than 0.05 was considered statistically significant otherwise specified.

## 3. Results

### 3.1. Characteristics of the Study Subjects

The age and gender distribution were shown in Table 1. Significant difference of age distribution was observed between NG and NC (*P* < 0.05). The female-to-male ratio was more than 3.0 in the three groups, and no significant difference of gender distribution was found among the three groups (*χ*^2^ = 0.008, *P*=0.996). Clinical characteristics of PTC were shown in Supplementary [Supplementary-material supplementary-material-1]. In this study, 15.7% of the patients with PTC had a family history; 34.7% of the patients with PTC were at stage 0-I; and 18.4% of the PTCs had lymph node metastasis.

### 3.2. Genotype and Allele Distributions of the Three SNPs

Significant differences of genotype and allele distributions among the groups were found for all the three SNPs (all *P* < 0.05) (Table 2). For genotype distribution among the groups, chi-square test of linear-by-linear association was also performed, and significant trend associations were found for all the three SNPs (*P*_trend_ < 0.05). No departure from HWE was observed in NC group (for rs944289, *P*=0.669; for rs965513, *P*=0.127; and for rs1443434, *P*=0.974). Linkage disequilibrium between the three SNPs was analyzed using the online SNPStats program, and no significant linkage disequilibrium was found (*P* > 0.05) (Supplementary [Supplementary-material supplementary-material-1]). To make our results more dependable and avoid false-positive results, we also analyzed the difference of genotype and allele distributions with 1000 genome Han Chinese in Beijing (CHB) as controls, and the results were shown in Supplementary [Supplementary-material supplementary-material-1]. Except for rs1443434 (*P*_trend_=0.012), no significant distribution difference was found for rs944289 and rs965513 (*P* > 0.05).

### 3.3. Association between the Three SNPs and PTC/NG

We used codominant, dominant, recessive, overdominant, and allele models to evaluate associations of rs944289, rs965513, and rs1443434 polymorphisms with PTC and with NG (Tables 3–5). Because of the significant age difference between NG and NC, evaluation of associations between SNPs and NG was adjusted by age. Codominant model was detected as the best model for all the three SNPs in the analysis of associations between the SNPs and PTC risk, and recessive model was detected as the best model for all the three SNPs in the analysis of association between the SNPs and NG risk. For rs944289, T allele was associated with increased risks for both PTC (OR = 1.23, 95% CI: 1.08–1.41, *P*=0.002) and NG (OR = 1.28, 95% CI: 1.10–1.50, *P*=0.002). TT genotype significantly increased PTC risk (codominant model, OR = 1.52, 95% CI: 1.15–1.99, *P*=0.010) and NG risk (recessive model, OR = 1.60, 95% CI: 1.22–2.10, *P*=0.001); however, the risk effect of TT genotype on PTC was not significant after Bonferroni correction. For rs965513, A allele was associated with increased PTC risk (OR = 1.34, 95% CI: 1.08–1.67, *P*=0.007), and AA genotype significantly increased PTC risk under codominant model (OR = 3.81, 95% CI: 1.25–11.63, *P*=0.008). NG risk increased significantly for AA genotype under recessive model (OR = 4.53, 95% CI: 1.41–14.59, *P*=0.006). However, after Bonferroni correction, no association was still significant for rs965513. For rs1443434, G allele was associated with increased PTC risk (OR = 1.33, 95% CI: 1.10–1.61, *P*=0.003). Under the codominant model, GG genotype of rs1443434 significantly increased PTC risk (OR = 2.21, 95% CI: 1.15–4.23, *P*=0.010), but the association was not significant after Bonferroni correction. Powers were calculated under dominant and recessive models using Quanto 1.2.4 software on the basis of the sample size and minor allele frequencies in our study (Supplementary [Supplementary-material supplementary-material-1]). We also performed the association analyses with 1000 genome CHB as controls (Supplementary [Supplementary-material supplementary-material-1]–[Supplementary-material supplementary-material-1]). No genotype or allele on the three SNPs was found significantly associated with PTC (*P* > 0.05). For rs1443434, G allele was associated with increased risk of NG (OR = 1.87, 95% CI: 1.05–3.31, *P*=0.04), but the association was not significant after Bonferroni correction.

### 3.4. Cumulative Risk Effect of the Three SNPs on PTC

We performed chi-square test of linear-by-linear association and logistic regression to evaluate the combined effect of the three SNPs on PTC risk (Table 6). Total counts of risk alleles for each subject were calculated, with a range of 0–5. As a result, percentages of PTCs with more risk alleles (three, four, and five) were higher than that of NGs and NCs (*P*_trend_ < 0.001), and with the increasing of each risk allele, PTC risk increased 0.25-fold (OR = 1.25, 95% CI: 1.13–1.37, *P* < 0.001).

### 3.5. Haplotype Analysis

Haplotype analysis revealed that individuals carrying rs944289-rs965513-rs1443434 haplotypes T-G-G and T-G-T had increased risks of PTC (OR = 1.82, 95% CI: 1.25–2.64, *P*=0.002) and of NG (OR = 1.28, 95% CI: 1.06–1.54, *P*=0.011) (Supplementary [Supplementary-material supplementary-material-1]-[Supplementary-material supplementary-material-1]), respectively.

### 3.6. Association between the Three SNPs and PTC Risks after Stratified by Gender

Genotype and allele frequencies of the three SNPs in females and males are shown in Supplementary [Supplementary-material supplementary-material-1]. After stratified by gender, associations between the three SNPs and PTC risk under codominant, dominant, recessive, overdominant, and allele models are shown in Tables 7–9. Significant associations with PTC risks were found for rs944289 and rs965513 in females, and for rs1443434 in males. Codominant model was detected as the best model for all the significant comparisons. For rs944289, T allele (OR = 1.29, 95% CI: 1.10–1.50, *P*=0.001) and TT homozygote (codominant model: OR = 1.67, 95% CI: 1.22–2.28, *P*=0.005) significantly increased PTC risk in females, but the risk effect of TT genotype was not significant after Bonferroni correction. For rs965513, A allele (OR = 1.30, 95% CI: 1.01–1.67, *P*=0.040) and AA homozygote (codominant model: OR = 4.37, 95% CI: 0.92–20.65, *P*=0.044) significantly increased PTC risk in females, but the associations were not significant after Bonferroni correction any more. No significant association was found in males for rs965513 and rs944289. For rs1443434, G allele (OR = 1.74, 95% CI: 1.17–2.59, *P*=0.005) and GG homozygote (codominant model: OR = 4.91, 95% CI: 1.03–23.51, *P*=0.015) significantly increased PTC risk in males; however, the associations were not significant after Bonferroni correction. No significant association was found in females for rs1443434.

## 4. Discussion

In our present study, we evaluated the effect of TITF1 rs944289 and TITF2 rs965513 and rs1443434 polymorphisms on both PTC and NG, and we found that polymorphisms of rs944289 and rs1443434 are associated with PTC and NG, and PTC risk increases with the number of risk alleles of the three SNPs. After stratified by gender, a significant association between rs944289 polymorphism and PTC risk was only observed in females. Moreover, haplotypes T-G-G and T-G-T (rs944289-rs965513-rs1443434) are risk haplotypes of PTC and NG, respectively.

The polymorphism rs944289 predisposes to PTC through PTC susceptibility candidate 3 (PTCSC3) gene. PTCSC3 is a large intergenic noncoding RNA gene [25]. The risk allele of SNP rs944289 suppresses PTCSC3 by destroying a transcription factor-binding site in the promoter of PTCSC3 [26]. Our results showed that rs944289 polymorphism was associated with PTC risk, but no similar result was obtained when we used 1000 genome CHBs as controls, and we speculated that sample size was the main reason to the difference. In 1000 genome online database, only 45 CHBs was genotyped for the three SNPs, whereas in our own case-control study, 896 Han Chinese individuals were included. Moreover, geographical difference between 1000 genome CHBs (Beijing) and our controls (mainly from the Northeast China) may influence on these results. After stratified by gender, a significant association between rs944289 polymorphism and PTC risk was only observed in females, validating that the sex difference is involved in PTC pathogenesis. However, no statistically significant association between rs944289 and the risk of female breast cancer was also observed in a Chinese population [27], suggesting that rs944289 polymorphism may be required for female PTC specially, rather than breast cancer.

Although several meta-analyses have been performed to explore the association between these SNPs and thyroid cancer susceptibility, they still get no consistent result. Study of Chen et al. revealed the rs965513 polymorphism is significantly associated with risk of differentiated thyroid carcinoma (DTC) in Caucasians, but not in East Asians. The rs944289 polymorphism is associated with DTC in both Caucasians and East Asians [28]. Kang et al.'s study showed that the polymorphisms of rs944289 and rs965513 are not associated with thyroid cancer risk, but the rs1443434 polymorphism was significantly associated with thyroid cancer risk in overall population [29]. In Zhuang et al.'s study, rs965513 A allele is significantly associated with increased risk of thyroid cancer in both Caucasians and Asians [30]. In Gao et al.'s study, significant associations are found between the polymorphisms of rs944289 and rs965513 and risk of PTC risk in Caucasians and Asians [31]. In Figlioli et al.'s study, using GWAS and meta-analysis approaches confirms rs965513 as a risk locus of DTC [32].

Our present study focused on associations of the three SNPs (rs944289, rs965513, and rs1443434) and PTC risk in northern Chinese Han populations, whose ethnic background is different from the previous studies [16–18]. As a hospital-based case-control designed study, we must admit the potential selection bias which is common in such studies. In addition, sample size of our study (861 PTCs, 562 NGs, and 896 NCs) is relatively small, influencing the statistical power. Besides, environmental factors, such as smoking and drinking, are also remarkable risk factors for thyroid cancer, which were not analyzed in our study. Furthermore, we only selected three SNPs of interest for analysis, not taking other molecular subtyping into account. BRAF^V600E^ mutation, for example, which is an important mutation in thyroid cancer, may influence the genetic structure of the studied population, affecting the risk evaluation. Finally, studies on mechanism between rs944289 and rs1443434 polymorphisms and PTC should be performed to demonstrate the effect. Nevertheless, our study provides possible biomarkers for PTC diagnose and provides clews for further mechanism study, especially in northern Chinese Han populations.

## 5. Conclusions

The results of this study showed that polymorphisms of rs944289 and rs1443434 are associated with both PTC and NG in Chinese Han populations, and PTC risk increases with the number of total risk alleles of the three SNPs. Moreover, haplotypes T-G-G and T-G-T (rs944289-rs965513-rs1443434) are risk haplotypes of PTC and NG, respectively.

## Figures and Tables

**Table 1:** The age and gender distribution between PTCs, NGs, and NCs [*n* (%)].

Gender/age	PTC	NG	NC	*χ* ^*2*^/*F*	*P*
Age	43.7 ± 9.2	48.5 ± 10.0^∗^	43.7 ± 9.1	56.299	<0.001
Gender
Male	210 (24.4)	138 (24.6)	220 (24.6)	0.008	0.996
Female	651 (75.6)	423 (75.4)	676 (75.4)		

PTC, papillary thyroid carcinoma; NG, nodular goiter; NC, normal control; ^*∗*^NG vs. NC, *P* < 0.05.

**Table 2:** Genotype and allele distributions of the three SNPs in PTCs, NGs, and NCs.

Genotype/allele	PTC	NG	NC	*χ* ^2^	*P*	*P*_trend_
rs944289						
C/C	255 (29.8)	176 (31.7)	315 (35.5)	16.473	**0.002**	**0.003**
C/T	416 (48.7)	244 (43.9)	422 (47.6)			
T/T	184 (21.5)	136 (24.5)	150 (16.9)			
C	926 (54.2)	596 (53.6)	1052 (59.3)	12.821	**0.002**	
T	784 (45.8)	516 (46.4)	722 (40.7)			
rs965513						
G/G	647 (76.8)	449 (82.2)	704 (81.4)	16.768	**0.002**	**0.007**
A/G	181 (21.5)	85 (15.6)	157 (18.2)			
A/A	14 (1.7)	12 (2.2)	4 (0.5)			
G	1475 (87.6)	983 (90.0)	1565 (90.5)	8.181	**0.017**	
A	209 (12.4)	109 (10.0)	165 (9.5)			
rs1443434						
T/T	590 (70.7)	409 (78.5)	651 (76.1)	18.014	**0.001**	**0.003**
T/G	217 (26.0)	107 (20.5)	190 (22.2)			
G/G	28 (3.4)	5 (1.0)	14 (1.6)			
T	1397 (83.7)	925 (88.8)	1492 (87.3)	16.533	**<0.001**	
G	273 (16.3)	117 (11.2)	218 (12.7)			

*P*_trend_, *P* value of linear-by-linear association test. PTC, papillary thyroid carcinoma; NG, nodular goiter; NC, normal control. Significant *P* values in bold.

**Table 3:** Association between rs944289 polymorphism and PTC/NG.

Genotype/allele	PTC vs. NC			NG vs. NC		
	OR (95% CI)	*P*	AIC	OR (95% CI)^*∗*^	*P* ^*∗*^	AIC
Codominant						
C/C	1.00	0.010	2411.1	1.00	0.003	1835.4
C/T	1.22 (0.98–1.51)			1.06 (0.83–1.36)		
T/T	1.52 (1.15–1.99)			1.66 (1.22–2.26)		
Dominant						
C/C	1.00	0.011	2411.9	1.00	0.092	1842.5
C/T + T/T	1.30 (1.06–1.58)			1.22 (0.97–1.54)		
Recessive						
C/C + C/T	1.00	0.014	2412.4	1.00	**0.001**	1833.6
T/T	1.35 (1.06–1.71)			1.60 (1.22–2.10)		
Overdominant						
C/C + T/T	1.00	0.650	2418.1	1.00	0.240	1844.0
C/T	1.04 (0.87–1.26)			0.88 (0.70–1.09)		
Allele						
C	1.00	**0.002**	—	1.00	**0.002**	—
T	1.23 (1.08–1.41)			1.28 (1.10–1.50)		

^*∗*^Adjusted by age. PTC, papillary thyroid carcinoma; NG, nodular goiter; NC, normal control. Bonferroni correction was performed, and *P* < 0.003 was defined as statistical significance. Significant *P* values after Bonferroni correction in bold.

**Table 4:** Association between rs965513 polymorphism and PTC/NG.

Genotype/allele	PTC vs. NC			NG vs. NC		
	OR (95% CI)	*P*	AIC	OR (95% CI)^*∗*^	*P* ^*∗*^	AIC
Codominant						
G/G	1.00	0.008	2362.4	1.00	0.009	1795.4
G/A	1.25 (0.99–1.59)			0.82 (0.60–1.10)		
A/A	3.81 (1.25–11.63)			4.38 (1.36–14.11)		
Dominant						
G/G	1.00	0.021	2364.7	1.00	0.500	1802.4
G/A + A/A	1.32 (1.04–1.67)			0.91 (0.68–1.21)		
Recessive						
G/G + G/A	1.00	0.013	2363.9	1.00	0.006	1795.2
A/A	3.64 (1.19–11.10)			4.53 (1.41–14.59)		
Overdominant						
G/G + A/A	1.00	0.083	2367.1	1.00	0.140	1800.6
G/A	1.23 (0.97–1.57)			0.80 (0.59–1.08)		
Allele						
G	1.00	0.007	—	1.00	0.932	—
A	1.34 (1.08–1.67)			1.01 (0.78–1.32)		

^*∗*^Adjusted by age. PTC, papillary thyroid carcinoma; NG, nodular goiter; NC, normal control.

**Table 5:** Association between rs1443434 polymorphism and PTC/NG.

Genotype/allele	PTC vs. NC			NG vs. NC		
	OR (95% CI)	*P*	AIC	OR (95% CI)^*∗*^	*P* ^*∗*^	AIC
Codominant						
T/T	1.00	0.010	2339.3	1.00	0.370	1747.5
T/G	1.26 (1.01–1.58)			0.90 (0.68–1.18)		
G/G	2.21 (1.15–4.23)			0.53 (0.18–1.51)		
Dominant						
T/T	1.00	0.011	2340.1	1.00	0.320	1746.5
T/G + G/G	1.33 (1.07–1.65)			0.87 (0.67–1.14)		
Recessive						
T/T + T/G	1.00	0.022	2341.4	1.00	0.230	1746.1
G/G	2.08 (1.09–3.99)			0.54 (0.19–1.54)		
Overdominant						
T/T + G/G	1.00	0.070	2343.3	1.00	0.490	1747.0
T/G	1.23 (0.98–1.54)			0.91 (0.69–1.20)		
Allele						
T	1.00	**0.003**	—	1.00	0.230	—
G	1.33 (1.10–1.61)			1.16 (0.91–1.49)		

^*∗*^Adjusted by age. PTC, papillary thyroid carcinoma; NG, nodular goiter; NC, normal control. Bonferroni correction was performed, and *P* < 0.003 was defined as statistical significance. Significant *P* values after Bonferroni correction in bold.

**Table 6:** Cumulative risk alleles of the three SNPs and association with PTC.

No.	PTC	NG	NC	*χ* ^2^	*P*	*P*_trend_	OR (95% CI)^*∗*^	*P* ^*∗*^
0	146 (18.0)	108 (21.5)	178 (21.7)	29.881	**0.001**	**<0.001**	1.25 (1.13–1.37)	**<0.001**
1	298 (36.8)	194 (38.6)	338 (41.2)					
2	231 (28.5)	147 (29.3)	222 (27.1)					
3	111 (13.7)	44 (8.8)	76 (9.3)					
4	17 (2.1)	8 (1.6)	6 (0.7)					
5	7 (0.9)	1 (0.2)	0 (0.0)					
Total	810 (100.0)	502 (100.0)	820 (100.0)					

^*∗*^OR (95% *CI*) and *P* value were calculated by binary logistic regression for PTC vs. NC. *P*_trend_, *P* value of linear-by-linear association test. PTC, papillary thyroid carcinoma; NG, nodular goiter; NC, normal control. Significant *P* values in bold.

**Table 7:** Association between rs944289 polymorphism and PTC by gender.

Genotype/allele	Female			Male		
	OR (95% CI)	*P*	AIC	OR (95% CI)	*P*	AIC
Codominant						
C/C	1.00	0.005	1822.2	1.00	0.660	592.8
C/T	1.22 (0.95–1.57)			1.22 (0.80–1.86)		
T/T	1.67 (1.22–2.28)			1.09 (0.62–1.92)		
Dominant						
C/C	1.00	0.014	1824.7	1.00	0.410	591.0
C/T + T/T	1.34 (1.06–1.69)			1.18 (0.79–1.76)		
Recessive						
C/C + C/T	1.00	0.005	1822.8	1.00	0.930	591.6
T/T	1.48 (1.12–1.94)			0.98 (0.59–1.63)		
Overdominant						
C/C + T/T	1.00	0.970	1830.7	1.00	0.390	590.9
C/T	1.00 (0.81–1.25)			1.18 (0.81–1.73)		
Allele						
C	1.00	**0.001**	—	1.00	0.600	—
T	1.29 (1.10–1.50)			1.08 (0.82–1.42)		

Bonferroni correction was performed, and *P* < 0.003 was defined as statistical significance. Significant *P* values after Bonferroni correction in bold.

**Table 8:** Association between rs965513 polymorphism and PTC by gender.

Genotype/allele	Female			Male		
	OR (95% CI)	*P*	AIC	OR (95% CI)	*P*	AIC
Codominant						
G/G	1.00	0.044	1787.6	1.00	0.170	580.6
G/A	1.23 (0.93–1.61)			1.34 (0.82–2.19)		
A/A	4.37 (0.92–20.65)			3.23 (0.64–16.23)		
Dominant						
G/G	1.00	0.070	1788.7	1.00	0.130	579.7
G/A + A/A	1.28 (0.98–1.67)			1.44 (0.90–2.32)		
Recessive						
G/G + G/A	1.00	0.040	1787.8	1.00	0.150	580.0
A/A	4.19 (0.89–19.80)			3.04 (0.61–15.26)		
Overdominant						
G/G + A/A	1.00	0.170	1790.0	1.00	0.280	580.9
G/A	1.21 (0.92–1.59)			1.31 (0.80–2.13)		
Allele						
G	1.00	0.040	—	1.00	0.077	—
A	1.30 (1.01–1.67)			1.49 (0.97–2.28)		

**Table 9:** Association between rs1443434 polymorphism and PTC by gender.

Genotype/allele	Female			Male		
	OR (95% CI)	*P*	AIC	OR (95% CI)	*P*	AIC
Codominant						
T/T	1.00	0.170	1774.1	1.00	0.015	568.4
T/G	1.17 (0.90–1.51)			1.60 (1.01–2.53)		
G/G	1.78 (0.86–3.68)			4.91 (1.03–23.51)		
Dominant						
T/T	1.00	0.130	1773.5	1.00	0.013	568.5
T/G + G/G	1.21 (0.95–1.56)			1.75 (1.12–2.72)		
Recessive						
T/T + T/G	1.00	0.140	1773.3	1.00	0.038	570.5
G/G	1.71 (0.83–3.53)			4.37 (0.92–20.84)		
Overdominant						
T/T + G/G	1.00	0.300	1774.5	1.00	0.067	571.4
T/G	1.15 (0.89–1.48)			1.53 (0.97–2.41)		
Allele						
T	1.00	0.074	—	1.00	0.005	—
G	1.22 (0.98–1.52)			1.74 (1.17–2.59)		

## Data Availability

The data used to support the findings of this study are available from the corresponding author upon request.

## Supplementary Materials

Supplementary Materials Table S1: the PCR amplification primer sequence. Table S2: clinical characteristics of PTCs (*n* = 861). Table S3: linkage disequilibrium between the three SNPs. Table S4: genotype and allele distributions of the three SNPs in PTCs, NGs, and 1000genome CHBs. Table S5: summary of Quanto results for power calculation. Table S6: association between rs944289 polymorphism and PTC/NG with 1000genome CHB as controls. Table S7: association between rs965513 polymorphism and PTC/NG with 1000genome CHB as controls. Table S8: association between rs1443434 polymorphism and PTC/NG with 1000genome CHB as controls. Table S9: associations between TITF1/TITF2 haplotypes and risk of PTC. Table S10: associations between TITF1/TITF2 haplotypes and risk of NG. Table S11: genotype and allele frequencies of the three SNPs by gender (*n*, %).
