# Supplementary material for: Association of rs944289, rs965513, and rs1443434 in TITF1/TITF2 with Risks of Papillary Thyroid Carcinoma and with Nodular Goiter in Northern Chinese Han Populations
Source: Int J Endocrinol. 2020 Feb 11;2020:4539747. doi: 10.1155/2020/4539747 (PMC7036112; doi:10.1155/2020/4539747)
Supplement: Supplementary Materials — Table S1: the PCR amplification primer sequence. Table S2: clinical characteristics of PTCs (n = 861). Table S3: linkage disequilibrium between the three SNPs. Table S4: genotype and allele distributions of the three SNPs in PTCs, NGs, and 1000genome CHBs. Table S5: summary of Quanto results for power calculation. Table S6: association between rs944289 polymorphism and PTC/NG with 1000genome CHB as controls. Table S7: association between rs965513 polymorphism and PTC/NG with 1000genome CHB as controls. Table S8: association between rs1443434 polymorphism and PTC/NG with 1000genome CHB as controls. Table S9: associations between TITF1/TITF2 haplotypes and risk of PTC. Table S10: associations between TITF1/TITF2 haplotypes and risk of NG. Table S11: genotype and allele frequencies of the three SNPs by gender (n, %). [file 4539747.f1.docx]

| Table S1: The PCR amplification primer sequence. | |
| --- | --- |
| SNP | Primer Sequence (5' to 3') |
| rs965513 | F: ACGTTGGATGAGGCTCAGGTTATGTCTTTG |
|  | R: ACGTTGGATGATGAGTGGCTGGAATGGAAC |
| rs944289 | F: ACGTTGGATGAGCCTGTGAATGGACATTAG |
|  | R: ACGTTGGATGCTTGCAATTTAATTTGGTTG |
| rs1443434 | F: ACGTTGGATGCACCTCCTCCCGTTTACAGA |
|  | R: ACGTTGGATGACGTGGAAAAGACCGAGCAG |
| F, forward; R, reverse | |

| Table S2: Clinical characteristics of PTCs (n=861). | | |
| --- | --- | --- |
| Characteristic | n | % |
| Family history |  |  |
| Yes | 135 | 15.7 |
| No | 672 | 78.0 |
| Missing | 55 | 6.3 |
| Pathologic staging |  |  |
| 0-I | 299 | 34.7 |
| II | 14 | 1.6 |
| III | 24 | 2.8 |
| IV | 44 | 5.1 |
| Missing | 480 | 55.8 |
| Metastasis |  |  |
| Yes | 158 | 18.4 |
| No | 285 | 33.3 |
| Missing | 417 | 48.3 |
| PTC, papillary thyroid carcinoma | | |

| Table S3: Linkage disequilibrium between the three SNPs. | | | | | | | |
| --- | --- | --- | --- | --- | --- | --- | --- |
| SNPs | PTC vs NC | | |  | NG vs NC | | |
|  | *D'* | *r*^2^ | *P* |  | *D'* | *r*^2^ | *P* |
| rs944289-rs965513 | 0.0709 | 0.0008 | 0.0974 |  | 0.0712 | 0.0007 | 0.1543 |
| rs944289-rs1443434 | 0.0537 | 0.0006 | 0.1421 |  | 0.0923 | 0.0009 | 0.1201 |
| rs965513-rs1443434 | 0.0396 | 0.0011 | 0.0535 |  | 0.0166 | 0.0002 | 0.4509 |
| PTC, papillary thyroid carcinoma; NG, nodular goiter; NC, normal control | | | | | | | |

| Table S4: Genotype and allele distributions of the three SNPs in PTCs, NGs and 1000genome CHBs. | | | | | | |
| --- | --- | --- | --- | --- | --- | --- |
| Genotype/Allele | PTC | NG | 1000genome CHB | *χ*^2^ | *P* | *P*_trend_ |
| rs944289 |  |  |  |  |  |  |
| C/C | 255 (29.8) | 176 (31.7) | 11 (24.4) | 4.678 | 0.322 | 0.689 |
| C/T | 416 (48.7) | 244 (43.9) | 25 (55.6) |  |  |  |
| T/T | 184 (21.5) | 136 (24.5) | 9 (20.0) |  |  |  |
| C | 926 (54.2) | 596 (53.6) | 47 (52.2) | 0.186 | 0.911 |  |
| T | 784 (45.8) | 516 (46.4) | 43 (47.8) |  |  |  |
| rs965513 |  |  |  |  |  |  |
| G/G | 647 (76.8) | 449 (82.2) | 35 (77.8) | 7.843 | 0.069^*^ | 0.124 |
| A/G | 181 (21.5) | 85 (15.6) | 9 (20.0) |  |  |  |
| A/A | 14 (1.7) | 12 (2.2) | 1 (2.2) |  |  |  |
| G | 1475 (87.6) | 983 (90.0) | 79 (87.8) | 3.897 | 0.142 |  |
| A | 209 (12.4) | 109 (10.0) | 11 (12.2) |  |  |  |
| rs1443434 |  |  |  |  |  |  |
| T/T | 590 (70.7) | 409 (78.5) | 30 (66.7) | 16.676 | **0.004** | **0.012** |
| T/G | 217 (26.0) | 107 (20.5) | 13 (28.9) |  |  |  |
| G/G | 28 (3.4) | 5 (1.0) | 2 (4.4) |  |  |  |
| T | 1397 (83.7) | 925 (88.8) | 73 (81.1) | 14.968 | **<0.001** |  |
| G | 273 (16.3) | 117 (11.2) | 17 (18.9) |  |  |  |
| ^*^Fisher's exact test | | | | | | |
| *P*_trend_, *P* value of linear-by-linear association test | | | | | | |
| PTC, papillary thyroid carcinoma; NG, nodular goiter; CHB, Han Chinese in Beijing | | | | | | |
| Significant *P* values in bold | | | | | | |

| Table S5: Summary of Quanto results for power calculation. | | | | | | | | | |
| --- | --- | --- | --- | --- | --- | --- | --- | --- | --- |
| SNPs | PTC vs NC | | | |  | NG vs NC | | | |
|  | *OR*=1.4 | *OR*=1.6 | *OR*=1.8 | *OR*=2.0 |  | *OR*=1.4 | *OR*=1.6 | *OR*=1.8 | *OR*=2.0 |
| rs944289 |  |  |  |  |  |  |  |  |  |
| Recessive | 0.8196 | 0.9832 | 0.9994 | 0.9999 |  | 0.6994 | 0.9381 | 0.9927 | 0.9994 |
| Dominant | 0.8869 | 0.9918 | 0.9997 | 0.9999 |  | 0.8156 | 0.9773 | 0.9985 | 0.9999 |
| rs965513 |  |  |  |  |  |  |  |  |  |
| Recessive | 0.1294 | 0.2184 | 0.3294 | 0.4507 |  | 0.0956 | 0.1435 | 0.2019 | 0.2667 |
| Dominant | 0.8373 | 0.9867 | 0.9996 | 0.9999 |  | 0.6909 | 0.9337 | 0.9918 | 0.9993 |
| rs1443434 |  |  |  |  |  |  |  |  |  |
| Recessive | 0.1893 | 0.3412 | 0.5132 | 0.6733 |  | 0.1201 | 0.1939 | 0.2826 | 0.3777 |
| Dominant | 0.8878 | 0.9942 | 0.9999 | 0.9999 |  | 0.7417 | 0.9564 | 0.996 | 0.9998 |

| Table S6: Association between rs944289 polymorphism and PTC/NG with 1000genome CHB as controls. | | | | | | | |
| --- | --- | --- | --- | --- | --- | --- | --- |
| Genotype/Allele | PTC vs 1000genome CHB | | |  | NG vs 1000genome CHB | | |
|  | *OR* (95% *CI*) | *P* | *AIC* |  | *OR* (95% *CI*) | *P* | *AIC* |
| Codominant |  |  |  |  |  |  |  |
| C/C | 1.00 | 0.64 | 362.4 |  | 1.00 | 0.32 | 323.5 |
| C/T | 1.39 (0.67-2.88) |  |  |  | 1.64 (0.79-3.42) |  |  |
| T/T | 1.13 (0.46-2.79) |  |  |  | 1.06 (0.43-2.63) |  |  |
| Dominant |  |  |  |  |  |  |  |
| C/C | 1.00 | 0.43 | 360.7 |  | 1.00 | 0.30 | 322.8 |
| C/T+T/T | 1.31 (0.66-2.63) |  |  |  | 1.43 (0.71-2.89) |  |  |
| Recessive |  |  |  |  |  |  |  |
| C/C+C/T | 1.00 | 0.81 | 361.3 |  | 1.00 | 0.49 | 323.3 |
| T/T | 0.91 (0.43-1.93) |  |  |  | 0.77 (0.36-1.64) |  |  |
| Overdominant |  |  |  |  |  |  |  |
| C/C+T/T | 1.00 | 0.37 | 360.5 |  | 1.00 | 0.13 | 321.5 |
| C/T | 1.32 (0.72-2.41) |  |  |  | 1.60 (0.87-2.95) |  |  |
| Allele |  |  |  |  |  |  |  |
| C | 1.00 | 0.72 | -- |  | 1.00 | 0.81 | -- |
| T | 1.08 (0.71-1.65) |  |  |  | 1.05 (0.70-1.58) |  |  |
| PTC, papillary thyroid carcinoma; NG, nodular goiter; CHB, Han Chinese in Beijing | | | | | | | |

| Table S7: Association between rs965513 polymorphism and PTC/NG with 1000genome CHB as controls. | | | | | | | |
| --- | --- | --- | --- | --- | --- | --- | --- |
| Genotype/Allele | PTC vs 1000genome CHB | | |  | NG vs 1000genome CHB | | |
|  | *OR* (95% *CI*) | *P* | *AIC* |  | *OR* (95% *CI*) | *P* | *AIC* |
| Codominant |  |  |  |  |  |  | |
| G/G | 1.00 | 0.94 | 361.9 |  | 1.00 | 0.75 | 323.7 |
| G/A | 0.92 (0.43-1.95) |  |  |  | 1.36 (0.63-2.93) |  |  |
| A/A | 1.32 (0.17-10.33) |  |  |  | 1.07 (0.14-8.46) |  |  |
| Dominant |  |  |  |  |  |  |  |
| G/G | 1.00 | 0.88 | 360.0 |  | 1.00 | 0.47 | 321.7 |
| G/A+A/A | 0.95 (0.46-1.95) |  |  |  | 1.32 (0.63-2.76) |  |  |
| Recessive |  |  |  |  |  |  |  |
| G/G+G/A | 1.00 | 0.79 | 359.9 |  | 1.00 | 0.99 | 322.2 |
| A/A | 1.34 (0.17-10.46) |  |  |  | 1.01 (0.13-7.96) |  |  |
| Overdominant |  |  |  |  |  |  |  |
| G/G+A/A | 1.00 | 0.81 | 359.9 |  | 1.00 | 0.45 | 321.7 |
| G/A | 0.91 (0.43-1.93) |  |  |  | 1.36 (0.63-2.92) |  |  |
| Allele |  |  |  |  |  |  |  |
| G | 1.00 | 0.96 | -- |  | 1.00 | 0.54 | -- |
| A | 0.98 (0.52-1.87) |  |  |  | 1.22 (0.66-2.27) |  |  |
| PTC, papillary thyroid carcinoma; NG, nodular goiter; CHB, Han Chinese in Beijing | | | | | | | |

| Table S8: Association between rs1443434 polymorphism and PTC/NG with 1000genome CHB as controls. | | | | | | | |
| --- | --- | --- | --- | --- | --- | --- | --- |
| Genotype/Allele | PTC vs 1000genome CHB | | |  | NG vs 1000genome CHB | | |
|  | *OR* (95% *CI*) | *P* | *AIC* |  | *OR* (95% *CI*) | *P* | *AIC* |
| Codominant |  |  |  |  |  |  |  |
| T/T | 1.00 | 0.83 | 360.9 |  | 1.00 | 0.10 | 315.6 |
| T/G | 1.18 (0.60-2.30) |  |  |  | 1.66 (0.84-3.29) |  |  |
| G/G | 1.40 (0.32-6.18) |  |  |  | 5.45 (1.02-29.30) |  |  |
| Dominant |  |  |  |  |  |  |  |
| T/T | 1.00 | 0.57 | 358.9 |  | 1.00 | 0.08 | 315.1 |
| T/G+G/G | 1.20 (0.64-2.28) |  |  |  | 1.83 (0.95-3.51) |  |  |
| Recessive |  |  |  |  |  |  |  |
| T/T+T/G | 1.00 | 0.71 | 359.1 |  | 1.00 | 0.10 | 315.6 |
| G/G | 1.34 (0.31-5.81) |  |  |  | 4.80 (0.90-25.48) |  |  |
| Overdominant |  |  |  |  |  |  |  |
| T/T+G/G | 1.00 | 0.67 | 359.1 |  | 1.00 | 0.20 | 316.6 |
| T/G | 1.16 (0.60-2.25) |  |  |  | 1.57 (0.80-3.10) |  |  |
| Allele |  |  |  |  |  |  |  |
| T | 1.00 | 0.54 | -- |  | 1.00 | 0.04 | -- |
| G | 1.18 (0.70-2.01) |  |  |  | 1.87 (1.05-3.31) |  |  |
| PTC, papillary thyroid carcinoma; NG, nodular goiter; CHB, Han Chinese in Beijing | | | | | | | |

| Table S9: Associations between TITF1/TITF2 haplotypes and risk of PTC. | | | | | | | | |
| --- | --- | --- | --- | --- | --- | --- | --- | --- |
| Haplotype | rs944289 | rs965513 | rs1443434 | Frequency (%) | | | *OR* (95% *CI*) | *P* |
|  |  |  |  | PTC | NC | Total |  |  |
| 1 | C | G | T | 41.72 | 46.78 | 44.36 | 1.00 | --- |
| 2 | T | G | T | 32.28 | 32.02 | 32.12 | 1.13 (0.95-1.35) | 0.180 |
| 3 | C | G | G | 6.19 | 7.06 | 6.61 | 0.97 (0.67-1.40) | 0.860 |
| 4 | T | G | G | 7.37 | 4.61 | 5.95 | 1.82 (1.25-2.64) | **0.002** |
| 5 | C | A | T | 4.82 | 4.47 | 4.63 | 1.23 (0.80-1.89) | 0.340 |
| 6 | T | A | T | 4.82 | 3.97 | 4.37 | 1.35 (0.88-2.05) | 0.170 |
| 7 | C | A | G | 1.42 | 1.01 | 1.18 | 1.56 (0.58-4.18) | 0.380 |
| Rare | --- | --- | --- | 1.38 | 0.08 | 0.78 | 10.19 (0.19-545.86) | 0.250 |
| PTC, papillary thyroid carcinoma; NC, normal control; Rare, haplotypes with frequencies<0.01 | | | | | | | | |
| Significant *P* value in bold | | | | | | | | |

| Table S10: Associations between TITF1/TITF2 haplotypes and risk of NG. | | | | | | | | |
| --- | --- | --- | --- | --- | --- | --- | --- | --- |
| Haplotype | rs944289 | rs965513 | rs1443434 | Frequency (%) | | | *OR* (95% *CI*) | *P* |
|  |  |  |  | NG | NC | Total |  |  |
| 1 | C | G | T | 43.04 | 46.78 | 45.29 | 1.00 | --- |
| 2 | T | G | T | 37.35 | 32.02 | 34.14 | 1.28 (1.06-1.54) | **0.011** |
| 3 | C | G | G | 5.79 | 7.06 | 6.63 | 0.93 (0.62-1.39) | 0.720 |
| 4 | C | A | T | 4.12 | 4.47 | 4.35 | 0.86 (0.52-1.42) | 0.550 |
| 5 | T | G | G | 3.81 | 4.61 | 4.21 | 0.88 (0.54-1.44) | 0.620 |
| 6 | T | A | T | 4.26 | 3.97 | 4.04 | 1.33 (0.82-2.17) | 0.250 |
| Rare | --- | --- | --- | 1.63 | 1.09 | 1.33 | 1.36 (0.52-3.54) | 0.530 |
| *OR* (95% *CI*) and *P* values were adjusted by age | | | | | | | | |
| NG, nodular goiter; NC, normal control; Rare, haplotypes with frequencies<0.01 | | | | | | | | |
| Significant *P* value in bold | | | | | | | | |

| Table S11: Genotype and allele frequencies of the three SNPs by gender (n, %). | | | | | | | | | |
| --- | --- | --- | --- | --- | --- | --- | --- | --- | --- |
| SNPs | Gender | Group | n | Genotype | | |  | Allele | |
|  |  |  |  | 1/1 | 1/2 | 2/2 |  | 1 | 2 |
| rs944289 (C>T) | Female | PTC | 647 | 185 (28.6) | 313 (48.4) | 149 (23.0) |  | 683 (52.8) | 611 (47.2) |
|  |  | NC | 671 | 234 (34.9) | 324 (48.3) | 113 (16.8) |  | 792 (59.0) | 550 (41.0) |
|  | Male | PTC | 208 | 70 (33.7) | 103 (49.5) | 35 (16.8) |  | 243 (58.4) | 173 (41.6) |
|  |  | NC | 216 | 81 (37.5) | 98 (45.4) | 37 (17.1) |  | 260 (60.2) | 172 (39.8) |
| rs965513 (G>A) | Female | PTC | 633 | 489 (77.3) | 136 (21.5) | 8 (1.3) |  | 1114 (88.0) | 152 (12.0) |
|  |  | NC | 657 | 534 (81.3) | 121 (18.4) | 2 (0.3) |  | 1189 (90.5) | 125 (9.5) |
|  | Male | PTC | 209 | 158 (75.6) | 45 (21.5) | 6 (2.9) |  | 361 (86.4) | 57 (13.6) |
|  |  | NC | 208 | 170 (81.7) | 36 (17.3) | 2 (1.0) |  | 376 (90.4) | 40 (9.6) |
| rs1443434 (T>G) | Female | PTC | 635 | 454 (71.5) | 161 (25.4) | 20 (3.2) |  | 1069 (84.2) | 201 (15.8) |
|  |  | NC | 643 | 484 (75.3) | 147 (22.9) | 12 (1.9) |  | 1115 (86.7) | 171 (13.3) |
|  | Male | PTC | 200 | 136 (68.0) | 56 (28.0) | 8 (4.0) |  | 328 (82.0) | 72 (18.0) |
|  |  | NC | 212 | 167 (78.8) | 43 (20.3) | 2 (0.9) |  | 377 (88.9) | 47 (11.1) |
| 1/1, wild homozygote; 1/2, heterozygote; 2/2, mutant homozygote; 1, wild allele; 2, mutant allele; PTC, papillary thyroid carcinoma; NC, normal control | | | | | | | | | |
